# The Relationship Between Mortality, Nutritional Status, and Laboratory Parameters in Geriatric Chronic Obstructive Pulmonary Disease Patients

**DOI:** 10.7759/cureus.20526

**Published:** 2021-12-20

**Authors:** Ramazan Baldemir, Ali Alagoz

**Affiliations:** 1 Anesthesiology and Reanimation, University of Health Sciences, Ankara Atatürk Chest Diseases and Thoracic Surgery Training and Research Hospital, Ankara, TUR

**Keywords:** mean platelet volume, geriatric, chronic obstructive pulmonary disease, c-reactive protein to albumin ratio, c-reactive protein

## Abstract

Background: In geriatric patients, limitations in physical, mental, and/or social functions occur as a result of acute and/or chronic disease along with age-related degenerative changes. This study aimed to investigate the relationship between nutritional status, mean platelet volume (MPV), C-reactive protein (CRP)-to-albumin ratio (CAR), and mortality in geriatric chronic obstructive pulmonary disease (COPD) patients.

Methods: Patients aged 65 years and older who were tertiary state hospitalized with COPD were included in the study. Demographic data of the patients, diagnosis, nutritional risk score-2002 (NRS-2002) score, and body mass index (BMI) were recorded. Glucose, MPV, CRP, albumin, CAR values of the patients, as well as 30-day and 90-day mortality status after nutritional evaluation, were determined. Patients hospitalized for a reason other than COPD, those using anti-inflammatory drugs, patients with missing data, and those in intensive care units were excluded from the study. Patients were divided into two groups based on NRS-2002: NRS-2002; 1 and 2, and NRS-2002; ≥3.

Results: Four hundred eighteen patients were hospitalized for COPD. Of these patients, 279 were aged 65 and over, but due to missing data, only 261 patients' data were analyzed. The 30-day and 90-day mortality rates were quite high in patients with a diagnosis of COPD who needed nutritional support (37.5% and 49.8%). When the demographic data and laboratory values of the patients are examined according to the 30-day and 90-day mortality status, the MPV value is statistically significantly higher in those with mortality at the end of 90 days (p < 0.05). Despite the fact that the NRS-2002 ≥3 group had higher 30-day and 90-day mortality rates than the NRS-2002 1 or 2 groups, there was no statistically significant difference between the groups (p > 0.05).

Conclusions: As a result, 90-day mortality was observed in approximately half of the patients, and the majority of these patients were male. Parameters that could predict 30-day and 90-day mortality could not be determined without MPV. Inflammatory parameters such as MPV can guide the determination of nutritional needs, especially in geriatric patients with COPD. Because of the high mortality rates in geriatric patients with COPD who need nutritional support, nutritional support should be started without delay in these patients. There is a need for prospective randomized controlled multicenter studies on this subject.

## Introduction

Malnutrition is more common in geriatric patients because of their advanced age, cognitive decline, comorbid diseases, polypharmacy, depression, and poor appetite [1,2]. The presence of malnutrition in patients is associated with a prolonged hospital stay, increased morbidity, and mortality [3-5].

In geriatric patients, limitations in physical, mental, and/or social functions occur as a result of acute and/or chronic disease along with age-related degenerative changes. The risk of malnutrition increases due to advanced age, cognitive decline, comorbidities, polypharmacy, depression, and anorexia [1-3].

Nutritional disorders are common, especially in acute and chronic disease states, and malnutrition that occurs with the effects of the catabolic disease may cause an increase in mortality in elderly patients [4,6]. Therefore, it is important to carry out a nutritional risk assessment on all geriatric patients admitted to the hospital. For this purpose, nutritional risk assessment is performed in geriatric patients using body mass index (BMI), anthropometric measurements, and blood tests such as serum albumin and cholesterol values. For nutritional assessment, scoring systems such as the Subjective Global Assessment (SGA), mini nutritional assessment (MNA), geriatric nutritional risk index (GNRI), and nutritional risk score-2002 (NRS-2002) are employed [7-11].

Malnutrition is an important condition that is frequently seen in patients with a diagnosis of chronic obstructive pulmonary disease (COPD) and negatively affects morbidity and mortality [12]. The degree of chronic inflammation in patients with COPD is critical in the course of the disease [13]. In addition, considering that inflammation is an important factor for malnutrition in studies, it is recommended that data on inflammatory markers can be useful in the nutritional evaluation and testing their correlation with mortality [14]. C-reactive protein-to-albumin ratio (CAR) has been used as a prognosis and mortality indicator in many studies [15-17]. C-reactive protein (CRP) is indicated as an indicator of high inflammation, and low albumin is a negative phase reactant indicating malnutrition [15,16]. In addition, mean platelet volume (MPV) is thought to be associated with inflammation in many chronic diseases [18-21]. Therefore, CAR and MPV can be used as practical parameters for assessing mortality in patients hospitalized with the diagnosis of COPD.

The study aimed to investigate the relationship between nutritional status, laboratory parameters, and mortality in geriatric COPD patients hospitalized with COPD who were evaluated by the nutrition department of our hospital.

## Materials and methods

Following the approval of the Ethics Committee (Ankara Atatürk Chest Diseases and Thoracic Surgery Training and Research Hospital Scientific Studies Commission, Date: November 11, 2021, Number: 12), this retrospective study was conducted in a state hospital which is a tertiary chest diseases center by analyzing the nutrition unit records between January 2019 and December 2019. Patients aged 65 years and older who were hospitalized with COPD and who were consulted by the nutrition department were included in the study.

Demographic data of the patients, diagnosis, NRS-2002 score, and BMI were recorded. After nutritional examination, the patients' glucose, MPV, CRP, albumin, and CAR values, as well as their 30/90-day mortality status, were determined. Patients hospitalized for a reason other than COPD, those with a history of anti-inflammatory drug usage, those with missing data, and those in intensive care units were excluded from the study. CAR was calculated by dividing the CRP value by the albumin value. According to NRS-2002, patients were evaluated into two groups: NRS-2002; 1 and 2, and NRS-2002; ≥3.

Statistical analysis

Data analyses were performed by using SPSS for Windows, version 22.0 (SPSS Inc., Chicago, IL, United States). Whether the distribution of continuous variables was normal or not was determined by the Kolmogorov-Smirnov test. The Levene test was used for the evaluation of the homogeneity of variances. Continuous data were described as mean ± standard deviation for normal distributions and median (inter-quartile range) for skewed distributions unless otherwise specified. Categorical data were described as a number of cases (%). Statistical analysis differences in normally distributed variables between two independent groups were compared by the Student’s t-test. Statistical analysis differences in not normally distributed variables between two independent groups were compared by the Mann Whitney U test. Categorical variables were compared using Pearson’s chi-square test or Fisher’s exact test. Univariate and multivariate binary logistic regression analyses were performed to assess the association between 30-days and 90-days mortality and the risk factor findings. It was accepted that a p-value of 0.05 was a significant level in all statistical analyses.

## Results

**Figure 1:**
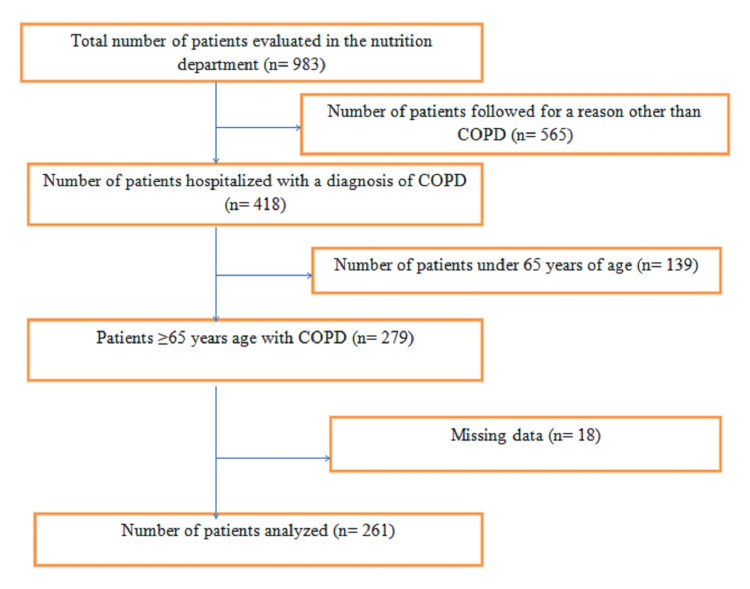
Flow chart of the patients. COPD: chronic obstructive pulmonary disease.

The data of 983 patients were evaluated by the nutrition department in 2019. Of these patients, 418 were hospitalized for COPD, and 279 were aged 65 and over. Eighteen patients were excluded due to missing data, and the data of 261 patients were analyzed (Figure 1).

The demographic data of the patients and their 30-day and 90-day mortality status are given in Table 1.

**Table 1:** Demographic data of the patients, initiation of nutritional support, 30-day and 90-day mortality rates. Continuous variables are expressed as mean ± standard deviation (SD) and categorical variables are expressed as frequency (percentage). BMI: body mass index.

	All patients (n:261)
Age, mean ± SD	76.03 ± 7.61
Gender, n (%)	
Male	174 (66.7%)
Female	87 (33.3%)
BMI (kg/m²), mean ± SD	23.94 ± 4.96
30-day mortality	98 (37.5%)
90-day mortality	130 (49.8%)

Age and MPV with a p-value of 0.25 were included in the multivariate analysis and in the univariate regression analysis to evaluate the factors that could predict 30-day and 90-day mortality based on demographic and laboratory data. However, as a result of multivariate regression analysis, a parameter that could predict 30-day and 90-day mortality was not detected (p > 0.05).

Despite the fact that the NRS-2002 3 group had higher 30-day and 90-day mortality rates than the NRS-2002 1 or 2 groups, there was no statistically significant difference between the groups (p > 0.05) (Table 2).

**Table 2:** Mortality rates of patients according to NRS-2002. Continuous variables are expressed as either the frequency (percentage). NRS: nutritional risk score.

	NRS-2002: 1 or 2 (n:23)	NRS-2002: ≥3 (n:238)	P-value
	n (%)	n (%)	
30-day mortality			0.461
No	16 (69.6%)	147 (61.8%)	
Yes	7 (30.4%)	91 (38.2%)	
90-day mortality			0.842
No	12 (52.2%)	119 (50.0%)	
Yes	11 (47.8%)	119 (50.0%)	

When the demographic data and laboratory values of the patients are examined according to the 30-day and 90-day mortality status, only the MPV value is statistically significantly higher in those with mortality at the end of 90 days (p < 0.05) (Table 3).

**Table 3:** Demographic data and laboratory values of patients according to 30-day and 90-day mortality status. Continuous variables are expressed as either the mean ± standard deviation (SD) or median (interquartile range) and categorical variables are expressed as frequency (percentage). BMI: body mass index, MPV: mean platelet volume, CRP: C-reactive protein, CAR: CRP-to-albumin ratio.

		30-day mortality		P-value	90-day mortality		P-value
		Yes	No		Yes	No	
Age		76.92±8.16	75.49±7.23	0.142	76.92±7.97	75.14±7.15	0.058
Gender	Male	62 (63.3%)	112 (68.7%)	0.366	87 (66.9%)	87 (66.4%)	0,930
	Female	36 (36.7%)	51 (31.3%)		43 (33.1%)	44 (33.6%)	
BMI (kg/m²)		24.31±5.16	23.72±4.84	0.346	24.33±4.94	23.55±4.97	0.204
MPV (fL)		9.40 (1.40)	9.10 (1.60)	0.061	9.40 (1.50)	9.10 (1.60)	0.030
Glucose (mg/dL)		119 (71)	115 (71)	0.541	119.50 (77)	112 (60)	0.285
Albumin (g/L)		27.30 (8.40)	28.80 (7.20)	0.293	28.15 (8.50)	28.70 (6.40)	0.289
CRP (mg/L)		97.17 (104.78)	79.18 (96.83)	0.185	86.92 (100.77)	86.14 (102.64)	0.394
CAR		3.19 (4.35)	2.63 (4.18)	0.206	2.96 (3.91)	2.78 (4.47)	0.497

## Discussion

In this study, mortality developed at the end of 90 days in approximately half of the predominantly male and geriatric patients with a mean age of 76.03 who were hospitalized with COPD and required a nutrition department evaluation. According to the multivariate regression analysis, a parameter that could predict 30-day and 90-day mortality could not be determined. However, the MPV value is significantly higher in patients with 90-day mortality. This study is special in that it included geriatric COPD patients, the majority of whom required hospitalization.

Due to the excess of comorbidities in geriatric patients and the existence of pharmacokinetic and pharmacodynamic differences, geriatric patients are handled differently from non-geriatric adult patients in many different clinical situations [22,23]. This shows that nutritional assessment should also be dealt with differently. For many reasons, malnutrition is more common in the elderly than in other age groups [1-3]. There is also a chronic inflammatory process in patients with a diagnosis of COPD, and this situation brings along a catabolic process in patients [13,24].

Regardless of the diagnosis of the disease, it is recommended that the elderly be screened for nutrition even if they are overweight or obese [1]. The validity of BMI as a measure of overweight and obesity decreases in geriatric patients due to changes in body composition and shortening of stature with aging [25-27]. In our study, there was no significant difference in BMI between the groups with and without 30-day and 90-day mortality.

When the catabolic process caused by inflammatory diseases is added to old age, the risk of malnutrition increases, and accordingly, there is an increase in morbidity and mortality [4,6,14,24]. In our study, mortality occurred in 37.5% of the patients after 30 days and 49.8% at the end of 90 days. The participants in our study did not include all geriatric patients with COPD who were hospitalized. It includes geriatric patients with COPD who were consulted by the nutrition department considering the need for nutritional support. Therefore, if there is malnutrition or risk of malnutrition in patients hospitalized with the diagnosis of COPD, it can be stated that mortality increases. This result shows that nutritional screening is very important, especially in geriatric patients with a COPD diagnosis, and that malnutrition must be prevented in this patient group. However, in the present study, no relationship was found between NRS-2002 and BMI and mortality. As a result of the clinician's observation, patients in need of nutritional support were identified. Therefore, we think that standard parameters are needed for the assessment of nutritional risk and mortality in geriatric patients.

CAR is used as an indicator of morbidity and mortality in many clinical situations in studies [15-17]. In the present study, we thought that CRP and CAR values could predict mortality in geriatric COPD patients in terms of showing the degree of inflammation. However, we found that CRP or CAR could not predict mortality in geriatric COPD patients. However, we think that multicenter and prospective studies should investigate the relationship between CAR and mortality in patients with geriatric COPD.

Another important result we obtained in our study is that MPV was higher in the group with mortality after 90 days in geriatric COPD patients. MPV is a parameter that is frequently used as an indicator of platelet function [18]. It has also been found to be associated with the inflammatory process in many chronic disease states, and it is stated that it reflects the inflammatory load and the severity of the disease [18-21,28,29]. It is assumed that MPV reflects platelet activity and could be a prognostic biomarker in the prediction of cardiovascular events [30,31]. It has also been reported in studies to be associated with various prothrombotic and proinflammatory diseases [32]. An increase in MPV levels has been found in cardiovascular disease, stroke, chronic respiratory diseases, chronic kidney failure, intestinal diseases, rheumatoid diseases, diabetes, and a variety of cancers [32]. In contrast, a reduction in MPV has been observed in tuberculosis, ulcerative colitis, systemic lupus erythematosus in adults, and different neoplastic diseases during disease exacerbation [32]. In another study, it was stated that high preadmission MPV levels were associated with increased in-hospital mortality in geriatric patients with myocardial infarction [30]. In our study, high mortality rates may be associated with high comorbidity rates and ischemic cardiac and cerebral events that may be caused by chronic respiratory problems in geriatric COPD patients. In addition, we think that chronic nutritional disorders in patients may contribute to this negative process. We think that it would be useful to consider the relationship between MPV and nutritional assessment and mortality with prospective studies.

There are some limitations to the present study. First, our study is retrospective and single-center. Because it is retrospective, we could not determine the scoring used in nutritional assessment, especially for geriatric patients such as MNA. Second, since this study included only geriatric patients consulted with the nutrition department, other geriatric patients were not included in the evaluation.

## Conclusions

As a result, 90-day mortality was observed in approximately half of the patients, and the majority of these patients were male. Parameters that could predict 30-day and 90-day mortality could not be determined without MPV. Inflammatory parameters such as MPV can guide the determination of nutritional needs, especially in geriatric patients with COPD. Because of the high mortality rates in geriatric patients with COPD who need nutritional support, nutritional support should be started without delay in these patients. There is a need for prospective randomized controlled multicenter studies on this subject.
